# Women’s Knowledge and Attitudes Regarding the Risk Factors and Warning Signs of Breast Cancer in the Eastern Province, Saudi Arabia: A Cross-Sectional Study

**DOI:** 10.7759/cureus.49463

**Published:** 2023-11-26

**Authors:** Loai Albinsaad, Mohammed Alessa, Jawaher I Alraihan, Mohammed A Albesher, Haidar A Alessa, Asma Almubarak

**Affiliations:** 1 Surgery, College of Medicine/King Faisal University, Al-Ahsa, SAU; 2 Medicine, King Faisal University, Al-Ahsa, SAU

**Keywords:** saudi arabia, eastern province, adult females, knowledge, breast cancer

## Abstract

Introduction

Breast cancer is the leading type of cancer in women globally, contributing significantly to cancer-related deaths. In Saudi Arabia, it ranks as the second most common cancer among women. Studies have highlighted a lack of awareness about breast cancer, negatively impacting breast self-examination practices. The Eastern Province has particularly high incidence rates. Knowledge of risk and protective factors can aid in prevention and early detection. While some studies show good awareness, this research aims to assess women's knowledge and attitudes about breast cancer risk factors and warning signs in the Eastern Province.

Methodology

This is a cross-sectional study conducted in Saudi Arabia's Eastern Province from June to October 2023 to assess breast cancer awareness among adult females. Data were collected via an online survey. Data were cleaned in MS Excel (Microsoft Corporation, Redmond, Washington) and analyzed in IBM SPSS Statistics for Windows, Version 23 (Released 2015; IBM Corp., Armonk, New York).

Results

Our study assessed breast cancer awareness in 697 participants in Saudi Arabia's Eastern Province. Most were aged 20-24 years (30.3%), married (49.2%), and Saudi nationals (97.6%), with higher education (59.4%). Significantly, 69.2% received education on breast cancer risk factors/signs. Some had personal breast cancer diagnoses (5.0%), and 31.7% had affected family members. Participants showed good awareness of various risk factors and warning signs. Significant associations were found between nationality, personal breast cancer diagnosis, and knowledge and attitudes about risk factors. Age, marital status, occupation, education on breast cancer, and personal breast cancer diagnosis influenced knowledge and attitudes about warning signs.

Conclusion

Our study findings indicate generally good awareness of breast cancer risk factors and warning signs among participants. Age, marital status, occupation, education on breast cancer, and personal breast cancer diagnosis influenced knowledge and attitudes about risk factors and warning signs. Educational efforts should target lesser-known risk factors to enhance prevention and early detection.

## Introduction

Worldwide, breast cancer is the most common type of cancer among women. It is the main contributor to female cancer-related death and disability. Breast cancer accounts for 23% of all newly diagnosed cancers, according to the World Health Organization (WHO), and over 1.4 million women worldwide are diagnosed with it each year [1]. In Saudi Arabia, breast and lung cancer are the second most common cancers among women, accounting for 10% of all cancer-related deaths [2]. Studies have reported insufficient awareness about breast cancer, its risk factors, and its early detection methods, negatively influencing the practice of breast self-examination among Saudi females. A lack of knowledge regarding breast cancer, its risk factors, and early detection techniques has been shown to negatively impact Saudi women's breast self-examination practices [3]. According to a previous study, the Eastern Province had the highest incidence of breast cancer, accounting for 26% of total cases and an age-specific incidence rate of 22 per 100,000 [4]. Lack of knowledge could result in a delayed presentation at a late stage when no treatment will be beneficial [5]. Knowing the risk factors and protective factors can help prevent and decrease the incidence of breast cancer. While many breast cancer risk factors cannot be avoided, some can be [6]. Age, age at menarche and menopause, family history, lifestyles, and use of oral contraceptives are all common risk factors seen in women with breast cancer [7]. Each of these elements, alone or in combination, has the potential to cause breast cancer. The burdens of morbidity and mortality associated with breast cancer can be significantly decreased by having adequate knowledge of the risk factors and early warning symptoms [8]. Protective factors include physical activity, weight control, an appropriate diet, encouraging breastfeeding, avoiding radiation, and unwelcome hormone intake [9]. However, a study done in Riyadh targeting private clinics concluded that patients had good knowledge of breast cancer risk factors and symptoms. The majority were also aware of screening methods for breast cancer [10]. This study aims to assess the level of women’s knowledge and attitudes regarding the risk factors and warning signs of breast cancer in the Eastern Province.

## Materials and methods

This cross-sectional study focused on females in the Eastern Province, Saudi Arabia, to evaluate their knowledge about breast cancer risk factors and warning signs. The study was conducted between June 2023 and October 2023. Google Forms were used to develop an online survey for data gathering. The general public, specifically women, was reached with the online poll and invited to participate. Two or more data collectors were chosen from each city in the eastern region to sign up as many participants as feasible. An online self-administered questionnaire was used to collect data, and before beginning to fill it out, participants were required to provide their agreement to participate in the study. The survey was divided into three portions. The first section asked about sociodemographic information such as age, gender, nationality, place of residence, level of education, and marital status. Based on the Breast Module of the Cancer Awareness Measure (Breast-CAM) [11], the second section evaluates the participant's level of knowledge of the risk factors for breast cancer, and the third section addresses warning signs. It was modified according to the aim of the study and our culture. The study was presented to specialists in General Surgery for improvement and approval. It was first formed in English and then translated into Arabic to be comprehensible for the targeted population. After the translation underwent grammatical and linguistic changes, it was approved following a review of the Arabic version by three distinct language specialists. A pilot study with a small (15-person) group of people was conducted to ensure that everyone was answering the questions correctly and had a good understanding. 

Data were collected using a self-administered questionnaire. The proposed questionnaire included questions about breast cancer risk factors and warning signs awareness, as well as personal information (demographic and general characteristics), including sex, nationality, age, and level of medical school. No participant’s name was used, but a location number was. The questionnaire had to be filled out by each participant on their own. It took between two and five minutes to complete. The questionnaire was taken from a similar study and modified by the authors.

The study's subjects are adult females from the Eastern Province who agreed to participate in the study during its period between June and October 2023 and have met the inclusion criteria. The inclusion criteria consist of being a female aged over 16 years old who lives in the Eastern Province of Saudi Arabia and has consented to participate in the study. Females from other cities were excluded from this study. The exclusion criteria consist of being a female from outside the Eastern Province, less than 16 years old, a female healthcare practitioner, or not consenting to participate in the study. The sampling technique utilized is convenient random sampling, where the questionnaire is disseminated via social media platforms, and the female population of the Eastern Province is invited to participate through an online link. The sample size was calculated using the formula n = z²pq/d². With a confidence level of 95%, an estimated proportion of 50%, and a 5% level of precision, the minimum sample size was calculated to be 385. However, we tried to collect more participants, and candidates were included to ensure the sufficiency and accuracy of the results.

According to the inclusion and exclusion criteria, the participants who fulfilled the criteria and agreed to the given consent were enrolled. Each subject anonymously filled out the questionnaire. The data were stored in a trusted place, and only approved personnel had access to these data. Privacy and confidentiality are priorities of this study, and the confidentiality of participants is ensured, as their identities remain unknown.

Data analysis was performed using IBM SPSS Statistics for Windows, Version 23 (Released 2015; IBM Corp., Armonk, New York). Frequency and percentages were used to display categorical variables. Minimum, maximum, mean, and standard deviation were used to present numerical variables. The chi-square test was used for comparison between variables. The level of significance was set at 0.05.

Ethical approval was taken from the ethical committee of King Faisal University (KFU). In addition, the purpose of the study was explained to the participants and their consent was obtained. Also, the participants were assured of the confidentiality of the survey, and their names were not used.

## Results

Our study assessed breast cancer risk awareness among 697 participants in Saudi Arabia's Eastern Province. As shown in Table 1, the majority of the participants, 211 (30.3%), were aged 20-24, 343 (49.2%) were married, and nearly all, 680 (97.6%), were Saudi nationals. A significant proportion, 414 (59.4%), had higher education. Most resided in Al-Ahsa, 432 (62.0%), and worked as students, 304 (43.6%), or housewives, 152 (21.8%). Notably, 482 (69.2%) had received education on breast cancer risk factors/signs. Additionally, 35 (5.0%) had been personally diagnosed with breast cancer, and 221 (31.7%) had a family member affected.

**Table 1:** Sociodemographic features of the participants assessed for breast cancer risk and warning signs.

		Frequency (n=697)	Percentage
Age	<16 years	5	0.7
	16-19 years	137	19.7
	20-24 years	211	30.3
	25-29 years	54	7.7
	30-39 years	111	15.9
	>40 years	179	25.7
Nationality	Non-Saudi	17	2.4
	Saudi	680	97.6
Marital status	Married	343	49.2
	Single	323	46.3
	Widowed	12	1.7
	Divorced	19	2.7
Educational status	Primary school	10	1.4
	Intermediate school	18	2.6
	Secondary school	255	36.6
	Higher education	414	59.4
Regions	Al-Ahsa	432	62.0
	Al Dammam	151	21.7
	Al Qatif	58	8.3
	Al Khabar	37	5.3
	Others	19	2.7
Occupation	Student	304	43.6
	Employee	135	19.4
	Retired	31	4.4
	Housewife	152	21.8
	Unemployed	55	7.9
	Health sector	5	.7
	Other	15	2.2
Educated on risk factors/signs of breast cancer	Yes	482	69.2
Ever diagnosed with breast cancer	Yes	35	5.0
Any family member having breast cancer	Yes	221	31.7

Table 2 shows participants' knowledge and attitudes regarding breast cancer risk factors. Proportions reveal that a substantial number agree or strongly agree that previous breast cancer history (73.9%), hormone replacement therapy (39.5%), alcohol consumption (64.8%), obesity (37.3%), family history of breast cancer (60.1%), late/no children (23.4%), early menarche (9.3%), late menopause (16.9%), and moderate physical exercise (19.7%) can influence breast cancer risk. These findings reflect participant awareness of these risk factors.

**Table 2:** Knowledge and attitude of the participants regarding risk factors of breast cancer. HRT: hormone replacement therapy

		Strongly Disagree	Disagree	Weakly Disagree	Weakly Agree	Agree	Strongly Agree
Previous history of breast cancer increases breast cancer risk	N	15	63	23	81	359	156
	%	2.2	9.0	3.3	11.6	51.5	22.4
Using HRT can increase breast cancer risk development	N	27	134	67	194	222	53
	%	3.9	19.2	9.6	27.8	31.9	7.6
Alcohol drinking increases the risk of breast cancer	N	9	77	33	127	314	137
	%	1.3	11.0	4.7	18.2	45.1	19.7
Obesity increases breast cancer risk	N	31	193	64	148	210	51
	%	4.4	27.7	9.2	21.2	30.1	7.3
Family history of breast cancer increases breast cancer risk	N	30	105	21	122	285	134
	%	4.3	15.1	3.0	17.5	40.9	19.2
Late/no children increase the risk of breast cancer	N	84	252	62	136	120	43
	%	12.1	36.2	8.9	19.5	17.2	6.2
Early menarche increases the risk of breast cancer	N	152	356	53	71	44	21
	%	21.8	51.1	7.6	10.2	6.3	3.0
Late menopause increases the risk of breast cancer	N	73	333	74	99	92	26
	%	10.5	47.8	10.6	14.2	13.2	3.7
Moderate physical exercise 5 times a week increases the risk	N	154	266	48	92	114	23
	%	22.1	38.2	6.9	13.2	16.4	3.3

Table 3 shows the participants' knowledge and attitudes concerning the warning signs of breast cancer. Proportions reveal that a majority recognized thickening/lumps in the breast (459, 65.9%), thickening/lumps in the armpits (67, 9.6%), bleeding/discharge from the nipple (447, 64.1%), nipple retraction (208, 29.8%), changes in nipple location (352, 50.5%), rash on the nipple (351, 50.4%), redness of breast skin (299, 42.9%), change in breast/nipple size (432, 62%), change in breast/nipple shape (428, 61.4%), pain in one breast (391, 56.1%), and dimpling of breast skin (316, 45.3%) as potential signs.

**Table 3:** Knowledge and attitude of the participants regarding warning signs of breast cancer.

		No	Don't Know	Yes
Thickening/lump in the breast could be a sign of breast cancer	N	99	139	459
	%	14.2	19.9	65.9
Thickening/lump under armpit could be a sign of breast cancer	N	27	134	536
	%	3.9	19.2	76.6
Bleeding/discharge from nipple could be a sign of breast cancer	N	83	167	447
	%	11.9	24.0	64.1
Nipple tightening could be a sign of breast cancer	N	186	303	208
	%	26.7	43.5	29.8
Change in nipple location can be a sign of breast cancer	N	98	247	352
	%	14.1	35.4	50.5
A rash on the nipple could be a sign of breast cancer	N	115	231	351
	%	16.5	33.1	50.4
Redness of breast skin could be a sign of breast cancer	N	154	244	299
	%	22.1	35.0	42.9
Change in breast/nipple size may be a sign of breast cancer	N	103	162	432
	%	14.8	23.2	62.0
Change in the shape of the breast/nipple may be a sign of breast cancer	N	103	166	428
	%	14.8	23.8	61.4
Pain in one of the breast/nipples could be a sign of breast cancer	N	171	135	391
	%	24.5	19.4	56.1
Dimpling of breast skin may be a sign of breast cancer	N	81	300	316
	%	11.6	43.0	45.3

Table 4 shows the associations between demographics and participants' knowledge and attitudes regarding breast cancer risk factors, focusing on significant p-values. Notable associations were found between nationality (p=0.043) and ever being diagnosed with breast cancer (p=0.004) with participants' knowledge and attitudes about risk factors. These findings suggest that nationality and a personal breast cancer diagnosis significantly impact participants' awareness and attitudes toward breast cancer risk factors. Other features do not significantly impact the knowledge and attitude of patients about risk factors of breast cancer.

**Table 4:** Associations between demographics and knowledge and attitude of the participants about risk factors of breast cancer.

			Knowledge and Attitude About Risk Factors of Breast Cancer		Sig. Value
			Poor	Good	
Age	16-19 years	N	34	108	0.406
		%	17.7%	21.4%	
	20-24 years	N	56	155	
		%	29.2%	30.7%	
	25-29 years	N	12	42	
		%	6.3%	8.3%	
	30-39 years	N	32	79	
		%	16.7%	15.6%	
	>40 years	N	58	121	
		%	30.2%	24.0%	
Nationality	Non-Saudi	N	1	16	0.043
		%	0.5%	3.2%	
	Saudi	N	191	489	
		%	99.5%	96.8%	
Regions	Al-Ahsa	N	127	305	0.403
		%	66.1%	60.4%	
	Al Dammam	N	34	117	
		%	17.7%	23.2%	
	Al Qatif	N	13	45	
		%	6.8%	8.9%	
	Al Khabar	N	12	25	
		%	6.3%	5.0%	
	Others	N	6	13	
		%	3.1%	2.6%	
Marital status	Married	N	105	238	0.266
		%	54.7%	47.1%	
	Single	N	81	242	
		%	42.2%	47.9%	
	Widowed	N	3	9	
		%	1.6%	1.8%	
	Divorced	N	3	16	
		%	1.6%	3.2%	
Educational status	Primary school	N	6	4	0.100
		%	3.1%	0.8%	
	Intermediate school	N	6	12	
		%	3.1%	2.4%	
	Secondary school	N	64	191	
		%	33.3%	37.8%	
	Higher education	N	116	298	
		%	60.4%	59.0%	
Occupation	Student	N	76	228	0.294
		%	39.6%	45.1%	
	Employee	N	35	100	
		%	18.2%	19.8%	
	Retired	N	13	18	
		%	6.8%	3.6%	
	Housewife	N	47	105	
		%	24.5%	20.8%	
	Unemployed	N	17	38	
		%	8.9%	7.5%	
	Other	N	4	16	
		%	2.1%	3.2%	
Ever educated on risk factors/signs of breast cancer	Yes	N	131	351	0.745
		%	68.2%	69.5%	
Ever diagnosed with breast cancer	Yes	N	17	18	0.004
		%	8.9%	3.6%	
Family history of breast cancer	Yes	N	56	165	0.374
		%	29.2%	32.7%	

Table 5 shows the relationships between participant demographics and their knowledge and attitudes regarding warning signs of breast cancer. Several significant associations were observed. Notably, aged 25-29 years (p<0.001), being more educated or aware (p=0.032), student (p<0.001), having been educated on risk factors/signs of breast cancer (p<0.001), and ever being diagnosed with breast cancer (p<0.001) were found to significantly influence participants' knowledge and attitudes about breast cancer warning signs. 

**Table 5:** Associations between demographics and knowledge and attitude of the participants about warning signs of breast cancer.

			Knowledge and Attitude Regarding Risk Factors of Breast Cancer		Sig. Value
			Poor	Good	
Age	16-19 years	N	28	114	<0.001
		%	16.0%	21.8%	
	20-24 years	N	37	174	
		%	21.1%	33.3%	
	25-29 years	N	15	39	
		%	8.6%	7.5%	
	30-39 years	N	30	81	
		%	17.1%	15.5%	
	>40 years	N	65	114	
		%	37.1%	21.8%	
Nationality	Non-Saudi	N	2	15	0.264
		%	1.1%	2.9%	
	Saudi	N	173	507	
		%	98.9%	97.1%	
Regions	Al-Ahsa	N	113	319	0.333
		%	64.6%	61.1%	
	Al Dammam	N	29	122	
		%	16.6%	23.4%	
	Al Qatif	N	16	42	
		%	9.1%	8.0%	
	Al Khabar	N	12	25	
		%	6.9%	4.8%	
	Others	N	5	14	
		%	2.9%	2.7%	
Marital status	Married	N	103	240	0.032
		%	58.9%	46.0%	
	Single	N	66	257	
		%	37.7%	49.2%	
	Widowed	N	2	10	
		%	1.1%	1.9%	
	Divorced	N	4	15	
		%	2.3%	2.9%	
Educational status	Primary school	N	6	4	0.062
		%	3.4%	0.8%	
	Intermediate school	N	5	13	
		%	2.9%	2.5%	
	Secondary school	N	68	187	
		%	38.9%	35.8%	
	Higher education	N	96	318	
		%	54.9%	60.9%	
Occupation	Student	N	54	250	<0.001
		%	30.9%	47.9%	
	Employee	N	35	100	
		%	20.0%	19.2%	
	Retired	N	10	21	
		%	5.7%	4.0%	
	Housewife	N	54	98	
		%	30.9%	18.8%	
	Unemployed	N	19	36	
		%	10.9%	6.9%	
	Other	N	3	17	
		%	1.7%	3.3%	
Ever educated on risk factors/signs of breast cancer	Yes	N	104	378	<0.001
		%	59.4%	72.4%	
Ever diagnosed with breast cancer	No	N	156	506	<0.001
		%	89.1%	96.9%	
	Yes	N	19	16	
		%	10.9%	3.1%	
Family history of breast cancer	Yes	N	53	168	0.641
		%	30.3%	32.2%	

## Discussion

Breast cancer is a global leading cause of female cancer-related deaths, including in Saudi Arabia, where it is the second most common cancer among women. Inadequate awareness impacts breast self-examination practices. Our study, "Women's Knowledge and Attitudes Regarding the Risk Factors and Warning Signs of Breast Cancer in the Eastern Province: Cross-Sectional Study," aims to address this issue and draws comparisons with prior studies in the field to enhance breast cancer awareness and early detection practices.

The demographic profile of our study aligns with certain aspects of previous literature on breast cancer awareness. Similar to prior studies, our sample was primarily composed of younger women (20-24 years), which could be attributed to their increased exposure to health education campaigns regarding breast cancer [12]. The higher education levels are consistent with findings indicating that education often correlates with better breast cancer knowledge, aligning with previous studies showing that awareness is related to education [13]. However, the predominance of Saudi nationals in our study differs from some literature that includes a more diverse demographic. Nonetheless, the impact of awareness campaigns on breast cancer education, reflected by the 69.2% educated participants, is a shared finding in many studies aiming to improve awareness and early detection.

Regarding the awareness of participants about the risk factors of breast cancer, our findings indicate a generally high awareness of established breast cancer risk factors among participants, aligning with prior research. However, gaps in knowledge regarding lesser-known risk factors like hormone replacement therapy, early menarche, and late menopause were observed. To improve prevention efforts, educating women about these less-discussed factors is crucial for a comprehensive understanding of breast cancer risk [14].

Participants' knowledge and attitudes concerning the warning signs of breast cancer provide valuable insights into the awareness levels of these critical indicators. The majority of participants recognized several key warning signs, including thickening or lumps in the breast, nipple changes, and pain in one breast. These results are consistent with prior research in the field, which has consistently shown that women tend to be aware of these prominent breast cancer warning signs [15-17].

However, it is worth noting that there were variations in awareness levels for certain signs. For instance, while a substantial proportion recognized changes in nipple location and breast size, awareness of less common signs like nipple tightening and dimpling of breast skin was comparatively lower. This suggests that there is room for improvement in educating women about these less frequently discussed warning signs. Educational campaigns can effectively enhance awareness levels about the warning signs of breast cancer [18].

The associations between demographics and participants' knowledge and attitudes about breast cancer risk factors were notable. Associations were found between nationality (p=0.043) and having ever been diagnosed with breast cancer (p=0.004) with participants' knowledge and attitudes about risk factors. These findings indicate that nationality and personal breast cancer diagnosis significantly impact participants' awareness and attitudes toward breast cancer risk factors. However, other demographic features did not significantly influence knowledge and attitudes.

Comparing our results with prior studies, our findings align with research that has shown that personal experiences, such as a breast cancer diagnosis, can significantly influence knowledge and attitudes. The association with nationality is an interesting finding and may be specific to our region. Further research could explore the cultural and social factors that underlie this association.

Our study also reveals the relationships between participant demographics and their knowledge and attitudes regarding breast cancer warning signs, which yielded noteworthy results. The influence of age on awareness aligns with existing research, as older women tend to have higher knowledge levels of breast cancer warning signs. This is consistent with findings by Linsell et al., who reported a similar age-related trend [19].

The impact of education on the awareness of breast cancer risk factors and warning signs echoes previous studies that emphasize the role of education in enhancing knowledge. This underscores the significance of targeted educational campaigns to raise awareness among different demographic groups.

The strong influence of a personal breast cancer diagnosis on knowledge and attitudes about warning signs highlights the importance of personal experiences in shaping awareness. The association between occupation and awareness is an intriguing aspect that requires further investigation, as it may have unique implications in the context of breast cancer awareness campaigns. Overall, these findings provide valuable insights for tailoring awareness initiatives to specific demographic characteristics, ultimately contributing to better breast cancer prevention and early detection efforts.

This study had a few limitations that affected its findings. Initially, the distribution of age was not equal. Therefore, we could not compare younger and older age groups in a broad context.

Furthermore, an online survey was used to collect the data. Thus, it is probable that some individuals provided inaccurate information on the questionnaire. Lastly, cross-sectional research is susceptible to confounding, selection, and information bias. It does not quantify cause and effect.

## Conclusions

Our study contributes to the growing body of knowledge on breast cancer awareness and underscores the need for continued awareness campaigns tailored to the specific needs of diverse populations. Our study findings showed that participants had good awareness of risk factors and warning signs. Significant associations were found between nationality, personal breast cancer diagnosis, and knowledge and attitudes about risk factors. Age, marital status, occupation, education on breast cancer, and personal breast cancer diagnosis influenced knowledge and attitudes about warning signs. By improving knowledge and attitudes about breast cancer, we can work towards earlier detection, better prevention, and ultimately, improved outcomes for women in the Eastern Province of Saudi Arabia.
